# Impact of catheter ablation for ventricular tachycardia on left ventricular ejection fraction in patients with structural heart disease

**DOI:** 10.1002/joa3.70042

**Published:** 2025-03-17

**Authors:** Ashish Sood, Samual Turnbull, Kasun De Silva, Ashwin Bhaskaran, Richard G. Bennett, Timothy G. Campbell, Liza Thomas, Saurabh Kumar

**Affiliations:** ^1^ Department of Cardiology Westmead Hospital Sydney New South Wales Australia; ^2^ Westmead Applied Research Centre University of Sydney Vancouver Sydney Australia; ^3^ Division of Cardiology University of British Columbia Vancouver British Columbia Canada; ^4^ Center for Cardiovascular Innovation Vancouver British Columbia Canada

**Keywords:** catheter ablation, echocardiography, left ventricular function, structural heart disease, ventricular tachycardia

## Abstract

**Background:**

Catheter ablation (CA) is efficacious for the treatment of ventricular tachycardia (VT) in patients with structural heart disease; however, heart failure contributes to long‐term mortality in this cohort. Whether CA worsens left ventricular function requires investigation.

**Methods:**

We retrospectively analyzed 142 consecutive patients with structural heart disease undergoing CA for VT. Pre‐ablation left ventricular ejection fraction (LVEF) was compared to LVEF postablation, predictors of change in LVEF were identified, and the relationship between change in LVEF and arrhythmic recurrence was assessed.

**Results:**

Patients with ischemic cardiomyopathy (ICM) had lower pre‐ablation LVEF than patients with non‐ischemic cardiomyopathy (NICM) (36.2 ± 14.3% vs. 50.8 ± 12.8%, *p* < 0.001). There was no statistically significant change in LVEF following ablation for patients with ICM (*p* = 0.45) or NICM (*p* = 0.75). Patients with pre‐ablation LVEF ≤20% experienced the largest recovery in LVEF, mean recovery 5.3% (95% CI: 0.6–10.1), *p* = 0.03, with LVEF recovery postablation similar in ICM and NICM patients (*p* = 0.69). Recovery of LVEF was associated with a decreased incidence of ventricular arrhythmia (VA) recurrence (*p* = 0.03) and an increased VA‐recurrence‐free survival (*p* = 0.04).

**Conclusion:**

CA for VT does not cause a decline in LVEF among patients with structural heart disease. The subset of patients with severely impaired LVEF may experience an increase in LVEF following ablation and an associated reduction in VA recurrence.

## Graphical abstract

This study investigates the left ventricular ejection fraction (LVEF) before and after catheter ablation for ventricular tachycardia in patients with structural heart disease. Patients with severely impaired pre‐ablation LVEF demonstrated mild restoration in LVEF following ablation. This improved LVEF was associated with a lower risk of arrhythmic recurrence

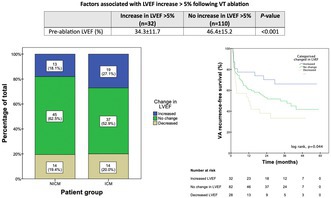

## 1 INTRODUCTION

Patients with structural heart disease (SHD) are at increased risk of ventricular tachycardia (VT) and sudden cardiac death.^1^ Although implantable cardioverter‐defibrillators (ICDs) terminate VT and reduce the risk of sudden cardiac death,^2^ recurrent VT and ICD therapy cause significant psychological distress for patients and impair quality of life; moreover, repeated ICD shocks increase mortality.^1^, ^3^, ^4^, ^5^ Catheter ablation (CA) for VT reduces the arrhythmic burden, reduces the risk of recurrent VT, improves quality of life, and can be lifesaving in VT storm.^5^

Heart failure exacerbations are a recognized complication of recurrent VT, contributing to over a third of the long‐term mortality following CA, and with an incidence rate that may exceed 10% per year.^6^, ^7^ Most commonly, CA of VT is performed with the application of radiofrequency energy to target tissue, causing thermal coagulative necrosis and rendering the arrhythmogenic substrate electrically inert.^8^ Owing to a paucity of contemporaneous data investigating the effect of CA on left ventricular function, it remains uncertain as to whether ablation of ventricular myocardium depresses ventricular function, predisposing patients to heart failure and worsening long‐term outcomes. In this study, we aimed to analyze how CA for VT affects left ventricular function in patients with SHD, and whether there was an additive effect from ablation on LV dysfunction.

## 2 METHODS

One hundred and forty‐two consecutive patients with SHD who had CA for VT at a single tertiary referral center (Westmead Hospital, Sydney, Australia) between November 2017 and December 2020 were retrospectively identified. Patients were excluded from analysis if they were under the age of 18, underwent CA for premature ventricular complexes, had a diagnosis of idiopathic VT (based on previous cardiac MRI), or had no pre‐ablation or postablation transthoracic echocardiogram (TTE). SHD was divided into ischemic cardiomyopathy (ICM) and non‐ischemic cardiomyopathy (NICM), distinguished by the presence or absence of significant coronary artery disease on coronary angiography. NICMs were classified according to the European Society of Cardiology Working Group on Myocardial and Pericardial diseases,^9^ and included idiopathic dilated cardiomyopathy, hypertrophic cardiomyopathy, arrhythmogenic right ventricular cardiomyopathy, infiltrative cardiomyopathies, inflammatory cardiomyopathies (e.g., myocarditis), valvular heart disease, and congenital heart disease. Heart failure with reduced ejection fraction (HFrEF) was defined by a clinical syndrome with signs and symptoms caused by an LVEF ≤40% and corroborated by elevated natriuretic peptide levels and/or objective evidence of pulmonary or systemic congestion, as outlined by the Writing Committee of the Universal Definition of Heart Failure.^10^

Patients were initiated on drug therapy specific to HFrEF, specifically renin‐angiotensin system inhibitors, aldosterone antagonists, and beta‐blockers, as tolerated. For the purpose of this study, patients on a combination of all three were recorded to be on “triple therapy.”^11^ As the standard of care for HFrEF during the study period (2017–2020) did not include sodium–glucose cotransporter‐2 inhibitors (SGLT‐2 inhibitors), these medications were not universally utilized. After ablation, patients without a cardiac resynchronization defibrillator (CRT‐D), for whom it was clinically indicated, had a CRT‐D inserted or upgraded from a single‐ or a dual‐chamber ICD. All patients provided written informed consent for their procedure. Baseline patient characteristics, procedural data, complications, pre‐ and postablation left ventricular parameters, and follow‐up information were collected. This study was approved by the Western Sydney Local Health District Human Research Ethics Committee.

### 2.1 Preparation

Anti‐arrhythmic drugs were withheld five half‐lives prior to the CA, except for amiodarone and where urgent ablation was necessitated. In patients with ICDs, therapies were turned off prior to ablation; these therapies were reenabled following procedural completion. Ventricular pacing rate was not changed pre‐ or postablation in this study population.

The CA was performed under conscious sedation or general anesthesia, as previously described.^12^ Routine setup involved insertion of a decapolar catheter to the coronary sinus, a quadripolar catheter to the right ventricular (RV) apex (for atrial and ventricular monitoring and pacing, respectively), and the use of intracardiac echocardiography for catheter visualization. Following sheath insertion, systemic anticoagulation, with a target activated clotting time of >350 s, was achieved with intravenous unfractionated heparin before left ventricular (LV) access; if epicardial access was planned (based on suspicion of epicardial or intramural substrate), anticoagulation was commenced after epicardial access was obtained. Epicardial access was obtained via a percutaneous approach, or where pericardial adhesions were anticipated, through a surgical epicardial approach with a sub‐xiphoid pericardial window. Coronary angiography was performed prior to any epicardial ablation to avoid coronary artery injury. Phrenic nerve stimulation was excluded by high‐output pacing (10 mA and 9 ms output). Either trans‐septal (large curve Agilis; Abbott Medical, IL, USA) or retrograde aortic approaches were used to access the endocardial LV. Intracardiac electrogram monitoring was performed using the CardioLab EP Recording System (General Electric, Boston, MA, USA) with bandpass filtering performed between 30 and 500 Hz.

### 2.2 Induction protocol

Programmed electrical stimulation (PES) was performed commencing with a 400 ms drive train with up to four extrastimuli from at least two RV sites, starting at 300 ms and decrementing by 10 ms down to ventricular refractoriness.^12^ This was followed by burst RV pacing down to ventricular refractoriness from the RV apex. Where this failed to induce the arrhythmia, PES and burst RV pacing were repeated using the highest tolerated isoprenaline dose (up to 20 μg bolus and 40 μg/min infusion). Hemodynamic stability was provided with inotropic support and/or mechanical circulatory support where required. This induction protocol was repeated following ablation to assess for arrhythmia inducibility.

### 2.3 Mapping

Cardiac 3‐dimensional (3D) electroanatomical mapping (EAM) was performed using either CARTO (Biosense Webster, Inc., Diamond Bar, CA, USA) or EnSite Precision (Abbott Medical) EAM systems. Chamber geometry, bipolar, and unipolar voltage maps were constructed during either sinus or paced rhythms. Where hemodynamically tolerated, activation maps of each VT were obtained. Bipolar, unipolar, and activation maps were collected using standard voltage criteria, with bipolar electrogram amplitudes classified as: dense scar <0.5 mV; scar 0.5–1.5 mV; normal >1.5 mV.^13^ Unipolar low voltage was defined as EGM amplitude <8.3 mV for LV^14^ and <5.5 mV for RV.^15^

### 2.4 Ablation

Ablation was performed using the SmartTouch Surround Flow irrigated, contact force sensing ablation catheter (Biosense Webster) or the TactiCath Sensor Enabled catheter (Abbott Medical). Ablation was performed with a contact force of ≥10 g, aiming for an impedance drop of ≥20 Ω and radiofrequency energy of up to 50 W. Where possible, intracardiac echocardiography was used to visualize the catheter tip and ensure adequate tissue contact. Lesions were delivered until the site was electrically unexcitable, with pacing at 10 mA at a 9 ms pulse width.^16^

Ablation was guided by substrate, activation, or pace mapping. Activation or entrainment mapping was used where the VT was hemodynamically tolerated, targeting presumptive isthmus and exits; where not tolerated or noninducible, a substrate‐based approach with pace mapping was used. The specific approach targeted presumptive channels and exits as determined by paced QRS morphology matched against the VT QRS morphology with a stimulus‐to‐QRS interval >40 ms, abnormal fractionated potentials, double potentials, late potentials during sinus and paced rhythm, and late abnormal ventricular activities.^17^

### 2.5 Definition of clinical VT and procedural success

The clinical VT was defined as any inducible VT with a 12‐lead electrocardiogram morphology and rate (within 20 ms) matching a VT documented to have occurred spontaneously before ablation. Nonclinical VTs were inducible VTs that did not meet either of these criteria. Where VT was inducible, procedural success was defined as: (1) Complete: no VT was inducible following ablation; (2) Partial: at least 1 clinical VT was noninducible following ablation but other VTs remained; or (3) Failure: the clinical VT remained inducible following ablation.

### 2.6 Echocardiographic assessment of LV parameters

A comprehensive and standardized TTE was performed on each patient prior to their procedure and 3 months postprocedure (median 137 days) by experienced medical professionals/sonographers, using commercially available ultrasound machines (Vivid E9, GE Healthcare, Chicago, IL, USA) at Westmead Hospital. In patients receiving a CRT‐D insertion or upgrade after ablation, this procedure was performed after the postablation TTE was performed. Images were acquired with patients in the left‐lateral decubitus position, from the parasternal, apical, and subcostal views using a 3.5‐MHz transducer. M‐mode, two‐dimensional, color, and Doppler images were obtained; three consecutive beats were saved in cineloop format, acquired at high frame rates (>55 fps). Measurements were performed according to the American Society of Echocardiography recommendations,^18^ using dedicated offline software (EchoPac 201, General Electric, Boston, MA, USA). The LV ejection fraction (LVEF) was calculated using the Simpson's biplane method. LV end‐diastolic diameter (LVEDD), LV end‐diastolic volume (LVEDV), LV end‐systolic diameter (LVESD), and LV end‐systolic volume (LVESV) were recorded. End‐diastole and end‐systole were defined by the frames in which the cardiac dimensions were largest and smallest, respectively. To assess for inter‐ and intra‐observer variability and reliability, 20 TTEs were rereported by two independent cardiologists at the study site.

### 2.7 Definition of improvement or decline in LVEF

Changes in LVEF were categorized as increased (>5% increase), decreased (>5% decrease), or unchanged (change of ≤5%).

### 2.8 Follow‐up

All patients were enrolled in a remote monitoring service for ICD monitoring, managed by Westmead Hospital. Any ICD therapies remotely identified prompted an in‐person follow‐up visit for further evaluation. Follow‐up data was obtained through a retrospective review of electronic hospital medical records, referring Cardiologist letters, and remote monitoring.

Recurrence of ventricular arrhythmia (VA) was defined as sustained VT ≥30 s or requiring ICD therapies (anti‐tachycardia pacing [ATP] or shocks) for VT or ventricular fibrillation following ablation. A 1‐month blanking period was imposed for VA recurrences to exclude nonfatal outcomes occurring before the actual performance of CA.

### 2.9 Statistical analysis

Statistical analysis was performed using Statistical Package for Social Sciences version 25 (IBM Corporation, Armonk, NY, USA). Continuous variables were expressed as mean ± SD or median (inter quartile range [IQR]) when significantly skewed and compared using independent samples *t*‐test for parametric data and Mann–Whitney U test for nonparametric data. Categorical variables were expressed as *n* (%). Utilizing statistical methodology recommended by previous guidelines,^19^ inter‐ and intra‐observer reliability was assessed using intraclass correlation coefficient (ICC), while variability was calculated as the mean of differences across ratings, expressed as mean ± SD. The ICC measures the proportion of variances explained by variation across raters: values <0.5 indicate poor reliability, 0.5–0.75 indicate moderate reliability, 0.75–0.9 indicate good reliability, and >0.9 indicate excellent reliability. Between‐subjects analysis was performed using Fisher's exact *t*‐test (or Chi‐square test where appropriate); within‐subjects analysis was performed using dependent samples *t*‐test for parametric data and Wilcoxon signed rank test for nonparametric data. A one‐way ANOVA was used to compare pre‐ablation LVEF among patients who experienced a change in LVEF following ablation. Two‐way mixed ANOVA was used to compare the mean differences in left ventricular ejection fraction (LVEF) following ablation for patients with ICM compared to NICM. To determine multivariate predictors of decline in LVEF following ablation, characteristics identified as potentially significant on univariate analysis (*p* < 0.1) were entered into a binomial logistic regression model. Time to VA recurrence and/or death was analyzed using Kaplan–Meier analysis, with a 1‐month blanking period for VA recurrence only. Log‐rank Chi‐square testing assessed differences in groups according to change in LVEF following ablation. A 2‐sided *p*‐value of <0.05 was considered to be statistically significant.

## 3 RESULTS

### 3.1 Baseline characteristics

One hundred and forty‐two patients meeting the inclusion criteria were included in this study. Seventy two (50.7%) were ablations for patients with NICM, and 70 (49.3%) were ablations for patients with ICM. The etiology of NICM was idiopathic dilated cardiomyopathy in 34 (47.2%), cardiac sarcoidosis in 11 (15.3%), arrhythmogenic right ventricular cardiomyopathy in 9 (12.5%), postmyocarditis in 4 (5.6%), hypertrophic cardiomyopathy in 4 (5.6%), congenital heart disease in 4 (5.6%), amyloid in 3 (4.2%), and valvular in 3 (4.2%). For the ablations in patients with ICM, the infarct territory was anterior in 18 (25.7%), inferior in 17 (24.3%), lateral in 3 (4.3%), and multi‐vessel in 32 (45.7%). The mean time from infarct to CA was 16.2 ± 10.1 years. Baseline characteristics for both groups of patients are outlined in Table 1. Patients with ICM tended to be older, have more traditional cardiovascular risk factors, and had a higher incidence of HFrEF.

**TABLE 1:** Baseline characteristics.

Characteristics	ICM (*n* = 70)	NICM (*n* = 72)	*p*‐value
Age (years)	70.3 ± 9.7	58.1 ± 14.6	<0.001
Male gender	64 (91.4)	59 (81.9)	0.10
Hypertension	41 (58.6)	28 (38.9)	0.02
Hyperlipidemia	60 (85.7)	26 (36.1)	<0.001
Diabetes mellitus	28 (40)	10 (13.9)	<0.001
Atrial fibrillation	30 (42.9)	24 (33.3)	0.24
Chronic kidney disease	17 (24.3)	6 (8.3)	0.01
History of HFrEF	56 (80.0)	26 (36.1)	<0.001
Previous CABG	30 (42.9)	0 (0)	<0.001
Implanted devices pre‐ablation^a^			0.14
None	9 (12.9)	17 (23.6)	
Single‐chamber ICD	25 (35.7)	20 (27.8)	
Dual‐chamber ICD	21 (30)	22 (30.6)	
Dual‐chamber PPM	0 (0)	3 (4.2)	
CRT‐D	15 (21.4)	10 (13.9)	
VT ablation procedures received			0.83
1	36 (72.0)	43 (81.1)	
2	9 (18.0)	4 (7.5)	
3	4 (8.0)	4 (7.5)	
4	1 (2.0)	1 (1.9)	
5	0 (0)	1 (1.9)	
VT storm on presentation	28 (40)	20 (27.8)	0.12

*Note*: Values are expressed as mean ± SD, or n(%).

Abbreviations: CABG, coronary artery bypass grafting; CRT‐D, cardiac resynchronization therapy–defibrillator; HFrEF, heart failure with reduced ejection fraction; ICD, implanted cardioverter–defibrillator; ICM, ischemic cardiomyopathy; NICM, non‐ischemic cardiomyopathy; PPM, permanent pacemaker; VT, ventricular tachycardia.

^a^ All patients had a defibrillator inserted postprocedure.

In this study, 56 patients with ICM (80.0%) and 26 patients with NICM (36.1%) had HFrEF. The HFrEF‐specific therapies utilized in this subgroup of patients are outlined in Table 2. Of note, 12 (21.4%) patients with ICM and 6 (23.1%) patients with NICM qualifying for HFrEF therapy could tolerate “triple therapy” following ablation. Eight patients with ICM (14.3%) and 4 patients with NICM (15.4%) had cardiac resynchronization therapy defibrillator (CRT‐D) upgrades following ablation (after postablation TTE).

**TABLE 2:** HFrEF therapies in patients with HFrEF undergoing VT ablation pre‐ and postablation.

Characteristics	ICM (*n* = 56)	NICM (*n* = 26)	*p*‐value
Pre‐ablation HFrEF medications (class)			
ACEi/ARB/ARNI	33 (58.9)	18 (69.2)	0.37
Beta‐blocker	40 (71.4)	16 (61.5)	0.37
MRA	20 (35.7)	7 (26.9)	0.43
HF triple therapy	10 (17.9)	4 (15.4)	0.78
Postablation HFrEF medications (class)			
ACEi/ARB/ARNI	32 (57.1)	19 (73.1)	0.17
Beta‐blocker	44 (78.6)	16 (61.5)	0.11
MRA	24 (42.9)	9 (34.6)	0.48
HF triple therapy	12 (21.4)	6 (23.1)	0.87

*Note*: Values are expressed as n(%).

Abbreviations: ACEi, angiotensin‐converting enzyme inhibitor; ARB, angiotensin receptor blocker; ARNI, angiotensin receptor‐neprilysin inhibitor; HF, heart failure; HFrEF, heart failure with reduced ejection fraction; ICM, ischemic cardiomyopathy; MRA, mineralocorticoid receptor antagonist; NICM, non‐ischemic cardiomyopathy; VT, ventricular tachycardia.

### 3.2 Procedural characteristics

The VT was inducible in 129 (90.8%) patients, and of those with inducible VT, the procedure was a complete success in 68 (47.9%), a partial success in 52 (36.6%), and unsuccessful in 9 (6.3%). There were no significant differences in procedural success, radiofrequency time, procedure time, and fluoroscopy time between ICM and NICM patients.

### 3.3 Outcomes and follow‐up

The mean follow‐up time for patients from ablation was 1211 ± 420 days. None of the patients undergoing VT ablation had postprocedure myocardial infarction. Major procedural complications occurred in 9 patients (6.3%), including 4 cases (2.8%) of anticipated complete atrioventricular block following ablation in the basal septum, 2 cases (1.4%) of pericardial bleeding (one requiring percutaneous drainage, one requiring surgical repair), 2 cases (1.4%) of retroperitoneal bleeding (one managed with surgical repair, one managed conservatively), and 1 case (0.7%) of femoral thrombosis related to mechanical circulatory support. VAs recurred in a total of 72 patients, with a median recurrence time of 109 days (IQR 55–372 days), excluding those occurring within the one‐month blanking period. However, 47.9% of VA recurrences were within the first 3 months. There were 18 deaths in this cohort, occurring at a mean of 647 ± 465 days.

### 3.4 Left ventricular parameters

**FIGURE 1:**
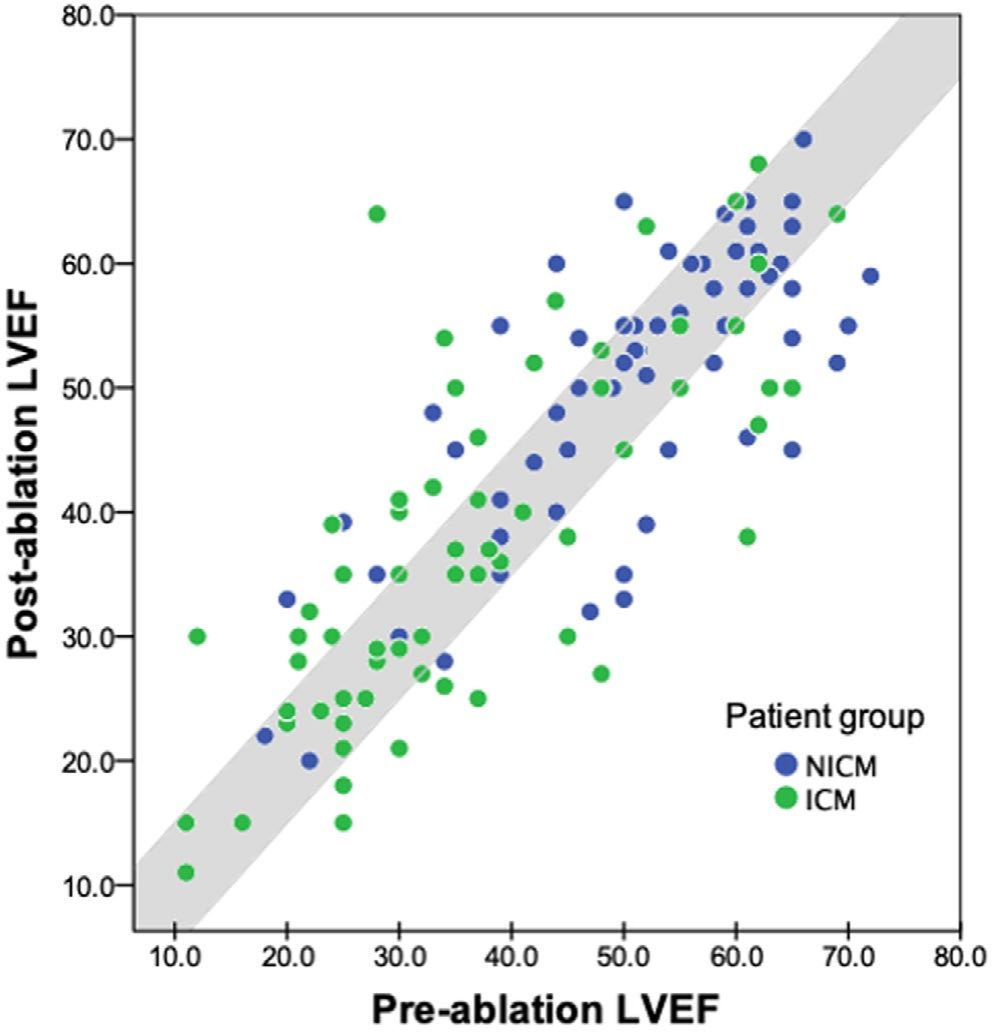
LVEF pre‐ablation compared to postablation among the entire cohort of NICM (blue dot) and ICM (green dot) patients. Each dot represents a patient and is situated according to their pre‐ and postablation LVEF. The gray region denotes a change in LVEF following ablation of ≤5%; dots outside this shaded area represent a rise or fall of LVEF >5% following ablation. Compared to patients with NICM, patients with ICM had a lower LVEF pre‐ablation (*p <* 0.001) and postablation (*p* < 0.001). The majority of patients (57.7%) with SHD had no change in LVEF, 22.5% had a >5% LVEF improvement, and 19.7% had a >5% LVEF decline.

**FIGURE 2:**
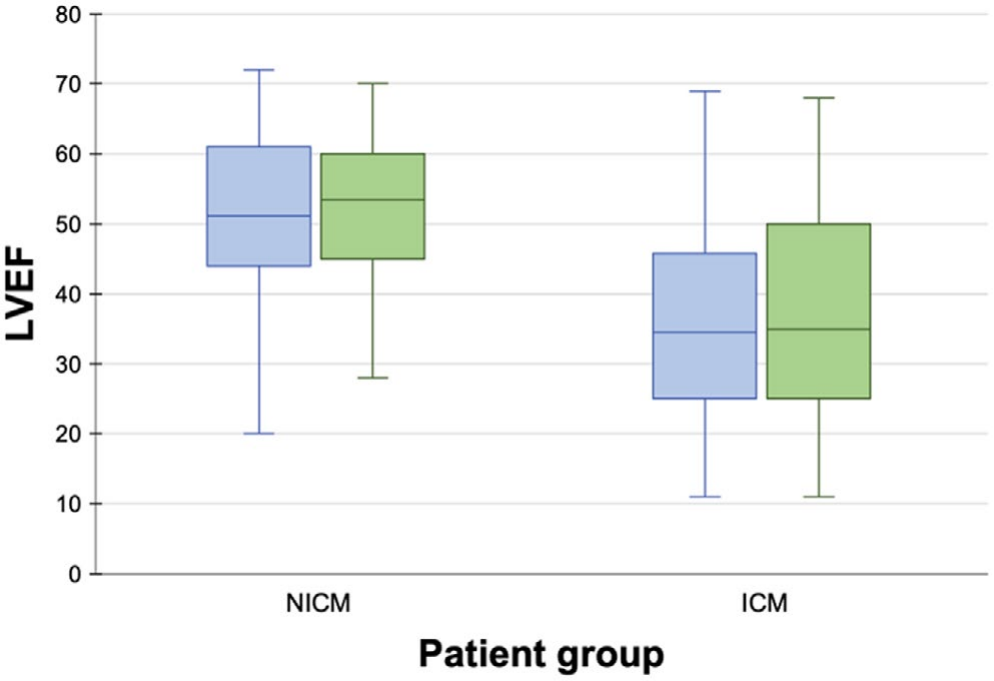
Comparison of pre‐ablation (blue bar) and postablation (green bar) LVEF for NICM and ICM patients. There was no significant difference in LVEF pre‐ versus postablation in either group.

**FIGURE 3:**
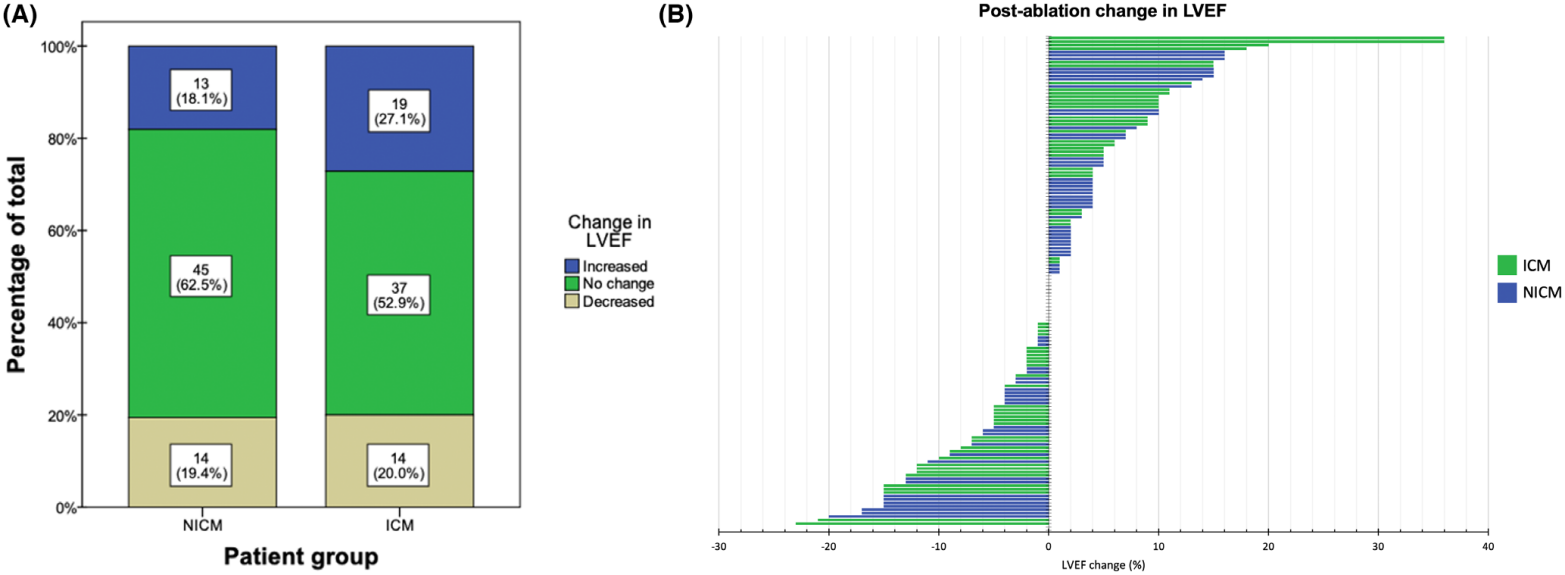
(A) Changes in LVEF following ablation for NICM and ICM patients. LVEF rise >5% in blue, LVEF decline >5% in brown, no significant change in green. Numbers are expressed in *n* (%). There were no significant differences in either group. (B) A case‐by‐case illustration of postablation change in LVEF for all patients. Blue bars are patients with NICM, while green bars are patients with ICM. Of the 14 patients with no net LVEF change postablation, 7 patients had NICM and 7 patients had NICM.

LVEF was calculated from TTE performed at a median of 14 days (IQR 3–69 days) pre‐ablation, and a median of 137 days (IQR 20–445 days) postablation. Pre‐ and postablation echocardiogram parameters are outlined in Table 3, and the distribution of pre‐ablation and postablation LVEF in patients in this study is depicted in Figure 1. Patients with ICM had lower pre‐ablation LVEF (36.2 ± 14.3 vs. 50.8 ± 12.8 in NICM, *p* < 0.001), and higher LVEDD, LVEDV, LVESD, and LVESV. There was no statistically significant change in LVEF following ablation for patients with ICM (*p* = 0.45) or NICM (*p* = 0.75), as depicted in Figure 2. After categorizing changes in LVEF into discrete categories, 82 of all patients with SHD (57.7%) had no change in LVEF, 32 (22.5%) had a >5% improvement in LVEF, and 28 (19.7%) had a >5% decline in LVEF. When compared between ICM and NICM patients, 37 (52.9%) versus 45 (62.5%), respectively, had no change in LVEF, 19 (27.1%) versus 13 (18.1%), respectively, had a >5% improvement in LVEF, and 14 (20.0%) versus 14 (19.4%), respectively, had a >5% decline in LVEF. This was not significantly different between these groups (*p* = 0.39), as depicted in Figure 3(A). A case‐by‐case illustration of postablation change in LVEF for all patients is depicted in Figure 3(B).

**TABLE 3:** Pre‐ and postablation echocardiogram parameters.

Characteristics	ICM	NICM	*p*‐value
Pre‐ablation			
LVEF (%)	36.2 ± 14.3	50.8 ± 12.8	<0.001
LVEDD (mm)	61.3 ± 10.0	53.8 ± 6.8	<0.001
LVEDV (mL)	189.2 ± 62.1	131.7 ± 41.3	<0.001
LVESD (mm)	50.7 ± 11.8	38.7 ± 9.4	<0.001
LVESV (mL)	130.8 ± 50.7	62.8 ± 32.9	<0.001
Postablation			
LVEF (%)	37.2 ± 14.2	51.2 ± 11.5	<0.001
LVEDD (mm)	57.6 ± 11.9	53.4 ± 7.1	0.03
LVEDV (mL)	190.0 ± 65.1	129.5 ± 46.9	<0.001
LVESD (mm)	47.2 ± 13.3	42.5 ± 14.1	0.10
LVESV (mL)	129.9 ± 64.3	65.5 ± 33.9	<0.001
Net change in LVEF			0.39
Increased (>5%)	19 (27.1)	13 (18.1)	
No change (± ≤5%)	37 (52.9)	45 (62.5)	
Decreased (>5%)	14 (20.0)	14 (19.4)	

*Note*: Values are expressed as mean ± SD, or *n* (%).

Abbreviations: ICM, ischemic cardiomyopathy; LVEDD, left ventricular end‐diastolic diameter; LVEDV, left ventricular end‐diastolic volume; LVEF, left ventricular ejection fraction; LVESD, left‐ventricular end‐systolic diameter; LVESV, left‐ventricular end‐systolic volume; NICM, non‐ischemic cardiomyopathy.

Furthermore, 17 (12.0%) of patients had >10% improvement in LVEF, while 19 (13.4%) had >10% decline in LVEF.

### 3.5 Predictors of postablation LVEF change

Predictors of a decrease in LVEF >5% following ablation on univariate analysis were pre‐ablation LVEF (*p* = 0.01), fluoroscopy time (*p* = 0.03), the patient taking an angiotensin‐converting enzyme (ACE‐i)/angiotensin receptor blocker (ARB)/angiotensin receptor‐neprilysin inhibitor (ARNI) pre‐ablation (*p* = 0.03), and septal RV ablation (*p* = 0.02). Full results of variables assessed on univariate analysis are shown in Table S1. The multivariate model results are outlined in Table 4. Pre‐ablation LVEF (*p* = 0.003) and pre‐ablation lack of ACE/ARB/ARNI administration (*p* = 0.004) remained significant independent predictors of LVEF decline following ablation. The only predictor of an increase in LVEF >5% following ablation was pre‐ablation LVEF (*p* < 0.001). Full results of variables assessed on univariate analysis are shown in Table S2. Procedural success was not a significant predictor of LVEF decline >5% (*p* = 0.17) or LVEF increase >5% (*p* = 0.62).

**TABLE 4:** Multivariate predictors of LVEF decline following ablation.

Variables	Odds ratio	95% confidence interval	*p*‐value
Pre‐ablation LVEF	1.107	1.036–1.183	0.003
Ablation in septal RV	0.728	0.126–4.194	0.32
Pre‐ablation use of ACE/ARB/ARNI	0.129	0.032–0.519	0.004

Abbreviations: ACEi, angiotensin‐converting enzyme inhibitor; ARB, angiotensin receptor blocker; ARNI, angiotensin receptor‐neprilysin inhibitor; LVEF, left ventricular ejection fraction; RV, right ventricle.

### 3.6 Influence of pre‐ablation LVEF on LVEF change following ablation

The pre‐ablation LVEF was compared among patients who experienced a >5% decrease, no change, or >5% increase in LVEF following ablation. Patients who experienced a >5% increase in LVEF following ablation had a lower pre‐ablation LVEF (34.3 ± 11.8) than patients who had no significant change (44.8 ± 15.3), which was lower still than patients who had a >5% decrease in LVEF (50.9 ± 14.3; *p* < 0.001 between groups). These changes remained significant when assessing NICM (*p* < 0.001) and ICM (*p* = 0.049) patients separately.

Exploratory analyses based on these findings identified subgroups of patients with LVEF impairment that derived an improvement in LVEF following ablation, with greater benefits realized by patients with more severe pre‐ablation LVEF impairment. SHD patients with pre‐ablation LVEF ≤20% had restoration of LVEF (mean increase 5.3%, 95% CI: 0.6–10.1, *p* = 0.03), as did those with LVEF 21%–50% (mean increase 2.5%, 95% CI: 0.2–4.7, *p* = 0.03). This was not significantly different between ICM and NICM patients (*p* = 0.69), indicating all patients with SHD realized these benefits. This remained true after excluding patients presenting with VT storm (*p* = 0.07) and patients with VA recurrence prior to postablation TTE (*p* = 0.99). VT storm on presentation was not significantly associated with LVEF improvement following ablation (*p =* 0.35). Further exploratory subgroup analysis within the LVEF 21%–50% group similarly showed a postablation LVEF increase in patients with more severely impaired pre‐ablation LV function; for example, those with LVEF 21%–35% had a mean LVEF increase of 4.9% (95% CI: 1.6–8.1, *p* < 0.01) while those with LVEF 36%–50% did not have a statistically significant change (mean increase 0.5%, 95% CI: −3.0–3.1, *p* = 0.97).

### 3.7 Predictors of VA recurrence

Predictors of VT recurrence following ablation on univariate analysis were patient age (*p* = 0.01), ICM (*p* = 0.03), septal RV ablation (*p* = 0.03), LVOT ablation (*p* = 0.04), postablation LVEF increase >5% (*p =* 0.01), and procedural success (*p* = 0.02). Full results of variables assessed on univariate analysis are shown in Table S3. The multivariate model results are outlined in Table S4. Patient age (*p* = 0.02), septal RV ablation (*p* = 0.01), postablation LVEF increase >5% (*p* = 0.01), and procedural success (*p* = 0.03 for complete, *p* = 0.03 for partial, *p* = 0.04 for unsuccessful) remained significant independent predictors of VA recurrence following ablation.

### 3.8 Changes in LVEF following ablation and VA recurrence‐free survival

**FIGURE 4:**
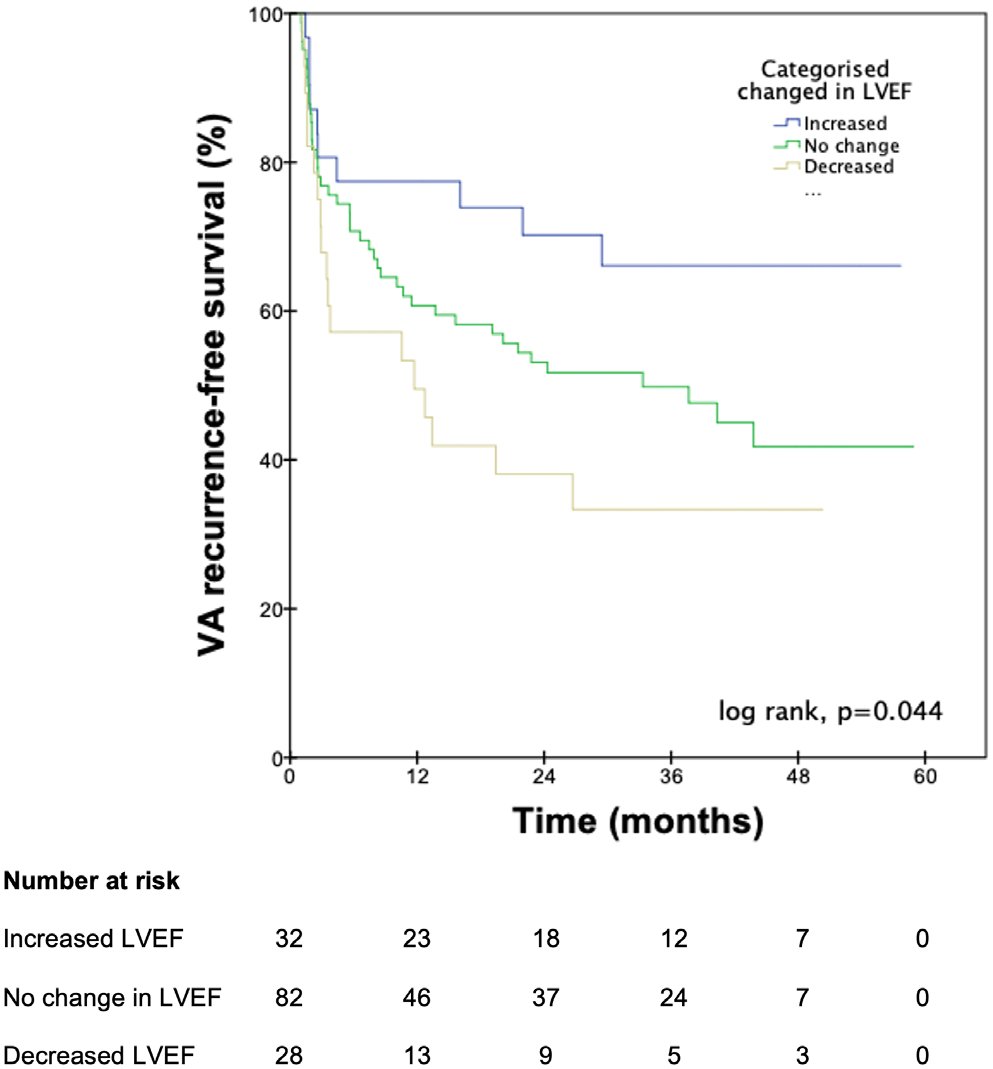
Kaplan–Meier curve of time to first VA recurrence following ablation, stratified by whether patients experienced a significant rise (>5%), decline (>5%), or no change in LVEF.

The incidence of VA recurrence was 31.3% in patients with LVEF improvement >5%, 52.4% in patients without significant LVEF change, and 64.3% in patients with LVEF decline >5%, which was statistically significant (*p* = 0.03). A Kaplan–Meier curve illustrating VA recurrence‐free survival following VT ablation is depicted in Figure 4, stratified by whether patients experienced an LVEF rise, LVEF decline, or neither following ablation. There were significant differences in VA‐recurrence‐free survival among the three groups (*p* = 0.04), with LVEF increase following ablation conferring a protective effect, while patients with LVEF decline following ablation experienced the worst outcomes. Although there was no statistically significant difference (*p* = 0.11) among the groups for a composite outcome of VA recurrence, death, or heart transplant, the trend lines favored those with LVEF improvement >5% with the best outcomes, followed by no change in LVEF and those with LVEF decline >5%.

### 3.9 Inter‐observer and intra‐observer reliability and variability

The mean inter‐observer variability was −0.9 ± 5.0, with an ICC of 0.98 indicating excellent reliability. The mean intra‐observer variability was 1.9 ± 5.5, with an ICC of 0.96 indicating excellent reliability.

## 4 DISCUSSION

In this retrospective analysis, we compared LVEF pre‐ab versus postablation in a consecutive cohort of patients with SHD and VT undergoing CA. Our main findings were:

1. Overall mean LVEF did not significantly change following CA of VT regardless of the underlying etiology of heart disease (ICM or NICM). Approximately 1/5th experienced a >5% improvement and another 1/5th experienced a 1/5th decline in LVEF, while 3/5th had no change in LVEF following ablation.
2. Independent predictors of LVEF decline following ablation were a higher pre‐ablation LVEF and lack of ACE/ARB/ARNi pre‐ablation.
3. LVEF change postablation was associated with VA recurrence, such that VA recurrence was lowest in patients with LVEF increase >5% and greatest in those with LVEF decline >5%.

The study demonstrates that VT ablation per se is not associated with a decline in ventricular function. Furthermore, the positive association of improvement in LVEF with subsequent outcomes (VA recurrence, death, or transplant) suggests that aggressive attempts should be made to optimize medical and device therapy for HFrEF postablation.

Heart failure is a common cause of long‐term mortality following CA for VT in patients with SHD. Heart failure‐related mortality ranges from 9.6% to 20% in previous studies over median follow‐up periods up to 41.5 months.^6^, ^20^, ^21^, ^22^, ^23^ Patients with lower LVEF have worse mortality following VT ablation.^24^ Indeed, LVEF is a key parameter in risk scores that predict acute hemodynamic deterioration or need for mechanical hemodynamic support during VT ablation (PAINESD score),^24^, ^25^, ^26^ early mortality following VT ablation,^27^ major complications following VT ablation,^28^ 1‐year incidence of VT recurrence, and all‐cause mortality after VT ablation (I‐VT score),^29^ and long‐term transplant or mortality after VT ablation (the Seattle Heart Failure Model).^30^, ^31^ This may be related to preexisting characteristics of patients requiring VT ablation (in our cohort, 57.8% had HFrEF, and 33.8% had previous VT storm), and repeated episodes of hypotension during the VT ablation (e.g. cardioversion‐related myocardial stunning, time spent in VT, use of general anesthesia).^7^ Furthermore, there is the potential for ablation itself to depress ventricular function through the replacement of contractile myocardium with scar.^7^

The impact of ablation on LVEF has been explored in previous studies;^3^, ^4^, ^13^, ^32^ however, the utility of their findings was limited by several factors: studies are over a decade old and predated contemporary medical therapy for HFrEF and technological advances in ablation,^13^, ^32^ had small sample sizes (range 6–62 patients),^13^, ^32^ or either primarily or wholly assessed LVEF change in patients with postinfarction VT.^3^, ^4^, ^13^, ^32^ To assess the impact of VT ablation across a broad spectrum of pre‐ablation LVEF, we included patients without initial LV impairment; as a result, we included patients with NICM (pre‐ablation LVEF 50.8 ± 12.8 vs. 36.2 ± 14.3 in ICM, *p* < 0.001). Furthermore, our ICM cohort had a higher mean pre‐ablation LVEF than historical studies (24%–30.7%).^3^, ^4^, ^13^, ^32^

Our study adds to the existing literature by recognizing the heterogeneity in LVEF change following ablation, when stratified by pre‐ablation LVEF. As a whole, there was no significant LVEF change in patients with SHD following ablation. Patients with impaired pre‐ablation LVEF, in particular, demonstrated mild restoration in LVEF following ablation, with increasing benefits realized by patients with more severely impaired pre‐ablation LVEF. While the statistically significant improvement in LVEF following ablation in patients without the most severe LV impairment is not clinically significant (mean increase 2.5% for patients with LVEF 21%–50%), it excludes a meaningful deleterious effect of ablation on LVEF. Notably, there was no significant difference between ICM and NICM patients in this regard.

NICM patients differ from ICM patients in several consequential ways: (1) substrate is more often intramurally/epicardially located; (2) VTs are less tolerated hemodynamically and are difficult to induce and access; and (3) theoretically, ablation involves contractile and viable myocardium, as opposed to ICM, where ablation generally involves regions of infarcted tissue that are minimally contractile or akinetic.^4^, ^7^, ^32^ It is therefore noteworthy that even in NICM patients, there was no significant decline in LVEF following ablation.

The postablation improvement in LVEF in patients with impaired LVEF in our study may be explained by the prevention of VA recurrence and its harmful impact on heart function. Heart failure generates arrhythmogenic substrate through complex mechanisms involving fibrosis and myocyte mechanical and electrical dysfunction, increasing the risk of VAs and sudden cardiac death.^33^ In turn, VAs predispose patients to worsening heart failure through ventricular dyssynchrony, myocyte metabolic alterations, and negative inotropy seen with many anti‐arrhythmics.^33^ This sets up a bidirectional negative feedback loop leading to worsening heart failure and recurring VA. CA for VT in selected patients interrupts this cycle, preventing further deterioration of ventricular function. For optimal management of such patients, the timely initiation of HFrEF therapies is required for reverse remodeling and myocardial function recovery. However, guideline‐directed medical therapies (GDMT) have historically been underutilized in eligible patients with HFrEF. Data from the CHAMP‐HF registry have demonstrated low real‐world uptake of GDMT—only 22.1% of patients were on GDMT, and only 1.1% of all patients were on target doses of all GDMT agents.^34^ This is similar to our study, where only 21.4%–23.1% of eligible patients with ICM and NICM were on triple therapy (the standard of care of HFrEF during our study period).

Improving LVEF is known to be negatively predictive of VA recurrence following VT ablation.^29^ Our study demonstrated that postablation changes in LVEF were predictive of both VA recurrence and time to VA recurrence. This adds to the literature for patients with VT, where previous studies for patients managed without ablation reported conflicting results.^35^, ^36^ However, the observational nature of this study prevents an assessment of the directionality of this relationship, whether the changes in LVEF following ablation caused the decrease in VA recurrence or vice versa. Notably, despite our study's finding that postablation LVEF improvement and decreased VA recurrence were significantly associated with each other, procedural success was only associated with the latter and not the former. Our study may have been underpowered for assessing the association between procedural success and LVEF improvement, with only 9 patients in our study having an LVEF ≤20% (the subset of patients experiencing the greatest increase in LVEF).

### 4.1 Limitations

This study was a single tertiary center retrospective study limiting its generalizability. Owing to the reliance on medical records and Cardiologist letters to obtain patients' medication regimens pre‐ and postablation, heart failure medication doses could not be reliably obtained. Additionally, TTEs were utilized to assess LV parameters in all patients. While this reflects real‐world practice, inherent limitations in tracing the endocardial border and incomplete visualization of the LV cavity in patients introduce the potential for error. To mitigate the effects of minor presumed changes in LVEF on TTE following ablation that may fall within the margin of error, this study employed a threshold of >5% change in LVEF for analyses. There were several limitations in LVEF assessment inherent to the retrospective study design, but these would reflect “real world” clinical practice. Lastly, owing to the small sample size, only certain pre‐ablation LVEF subgroups could be analyzed for postablation change in LVEF. Future studies with larger sample sizes would be beneficial in further stratifying within these groups.

## 5 CONCLUSIONS

This study demonstrated no significant decline in LVEF following VT ablation in patients with SHD, with no significant difference between patients with ICM and NICM. A subset of patients, characterized by severe LV impairment, may experience significant improvement in LVEF following ablation, and therefore a low LVEF should not preclude ablation therapy. Improvement in LVEF postablation is associated with better outcomes of VA recurrence and composite outcomes of VA recurrence, transplant, or mortality.

## CONFLICT OF INTEREST STATEMENT

There are no conflicts of interest to declare.

## DISCLOSURES

Dr. Saurabh Kumar is supported by the NSW Early Mid‐Career Fellowship. Dr. Timothy Campbell has received speakers' honoraria from Biosense Webster, Inc.

## ETHICS STATEMENT

The research protocol was approved through the Western Sydney Local Health District Human Research Ethics Committee.

## Supporting information


Data S1.
