# Supplementary material for: Impact of catheter ablation for ventricular tachycardia on left ventricular ejection fraction in patients with structural heart disease
Source: J Arrhythm. 2025 Mar 17;41(2):e70042. doi: 10.1002/joa3.70042 (PMC11912430; doi:10.1002/joa3.70042)
Supplement: Supplementary file 1 — Data S1. [file JOA3-41-e70042-s001.docx]

**Supplementary Table 1. Univariate predictors of LVEF decline following ablation.**

| Variables | Decline in LVEF >5% (n=28) | No decline in LVEF >5% (n=114) | *P*-value |
| --- | --- | --- | --- |
| Age (years) | 65.1±14.5 | 63.9±13.7 | 0.68 |
| Gender (male) | 26 (92.9%) | 97 (85.1%) | 0.37 |
| Ischemic cardiomyopathy | 14 (50.0%) | 56 (49.1%) | 0.93 |
| Previous CABG | 4 (14.3%) | 26 (22.8%) | 0.32 |
| Chronic kidney disease | 6 (21.4%) | 17 (14.9%) | 0.40 |
| Hypertension | 15 (53.6%) | 54 (47.4%) | 0.56 |
| Hyperlipidemia | 17 (60.7%) | 69 (60.5%) | 0.99 |
| Diabetes mellitus | 7 (25.0%) | 31 (27.2%) | 0.81 |
| Atrial fibrillation | 10 (35.7%) | 44 (38.6%) | 0.78 |
| History of HFrEF | 16 (57.1%) | 66 (57.9%) | 0.94 |
| Previous VT ablation | 8 (28.6%) | 46 (40.4%) | 0.25 |
| VT storm on presentation | 8 (28.6%) | 40 (35.1%) | 0.51 |
| Pre-ablation LVEF (%) | 50.9±14.3 | 41.9±15.1 | 0.01 |
| Pre-ablation LVEDD (mm) | 57.2±7.9 | 57.6±9.7 | 0.85 |
| Pre-ablation LVEDV (mL) | 163.0±45.4 | 166.2±64.7 | 0.86 |
| Pre-ablation LVESD (mm) | 44.8±10.7 | 45.0±12.7 | 0.95 |
| Pre-ablation LVESV (mL) | 96.0±57.1 | 105.2±55.8 | 0.68 |
| Pre-ablation ACEi/ARB/ARNI | 5 (17.9%) | 46 (40.4%) | 0.03 |
| Pre-ablation beta-blocker | 9 (32.1%) | 47 (41.2%) | 0.38 |
| Pre-ablation MRA | 5 (17.9%) | 22 (19.3%) | 0.86 |
| Pre-ablation ‘triple therapy’* | 0 (0.0%) | 14 (21.2%) | 0.06 |
| Post-ablation ACEi/ARB/ARNI | 6 (21.4%) | 45 (39.5%) | 0.08 |
| Post-ablation beta-blocker | 9 (32.1%) | 51 (44.7%) | 0.23 |
| Post-ablation MRA | 5 (17.9%) | 28 (24.6%) | 0.45 |
| Post-ablation ‘triple therapy’* | 1 (0.1%) | 17 (25.8%) | 0.17 |
| VT inducibility | 26 (92.9%) | 102 (89.5%) | 0.74 |
| Radiofrequency time (min) | 43.0±21.5 | 53.2±37.0 | 0.30 |
| Procedure time (min) | 224.3±67.4 | 247.3±89.1 | 0.34 |
| Fluoroscopy time (min) | 12.6±5.4 | 19.5±10.5 | 0.03 |
| Inferior LV ablation | 8 (28.6%) | 49 (43.0%) | 0.16 |
| Septal LV ablation | 14 (50.0%) | 41 (36.0%) | 0.17 |
| Anterior LV ablation | 6 (21.4%) | 21 (18.4%) | 0.72 |
| Lateral LV ablation | 2 (7.1%) | 11 (9.6%) | 1.00 |
| Inferior RV ablation | 5 (17.9%) | 8 (7.0%) | 0.13 |
| Septal RV ablation | 9 (32.1%) | 14 (12.3%) | 0.01 |
| Anterior RV ablation | 0 (0.0%) | 0 (0.0%) | - |
| Lateral RV ablation | 2 (7.1%) | 5 (4.4%) | 0.62 |
| LVOT ablation | 0 (0.0%) | 4 (3.5%) | 0.59 |
| RVOT ablation | 2 (7.1%) | 17 (14.9%) | 0.37 |
| Epicardial ablation | 1 (3.6%) | 10 (8.8%) | 0.69 |

*Values are expressed as n(%) for categorical variables, and mean ± SD for continuous variables. *Pre-ablation and post-ablation ‘triple therapy’ were analysed only in the eligible subgroup of patients with structural heart disease and heart failure with reduced ejection fraction (n=16 for decline in LVEF > 5%, n=66 for no decline in LVEF > 5%).*

**Abbreviations:** ACEi, angiotensin-converting enzyme inhibitor; ARB, angiotensin receptor blocker; ARNI, angiotensin receptor-neprilysin inhibitor; CABG, coronary artery bypass graft; HFrEF, heart failure with reduced ejection fraction; LV, left ventricle; LVOT, left ventricular outflow tract; LVEDD, left ventricular end-diastolic diameter; LVEDV, left ventricular end-diastolic volume; LVEF, left ventricular ejection fraction; LVESD, left-ventricular end-systolic diameter; LVESV, left-ventricular end-systolic volume; MRA, mineralocorticoid receptor antagonist; RV, right ventricle; RVOT, right ventricular outflow tract; VT, ventricular tachycardia.

**Supplementary Table 2. Univariate predictors of LVEF increase following ablation.**

| Variables | Increase in LVEF >5% (n=32) | No increase in LVEF >5% (n=110) | *P*-value |
| --- | --- | --- | --- |
| Age (years) | 65.5±10.3 | 63.7±14.7 | 0.43 |
| Gender (male) | 29 (90.6%) | 94 (85.5%) | 0.45 |
| Ischemic cardiomyopathy | 19 (59.4%) | 51 (46.4%) | 0.20 |
| Previous CABG | 10 (31.3%) | 20 (18.2%) | 0.11 |
| Chronic kidney disease | 7 (21.9%) | 16 (14.5%) | 0.32 |
| Hypertension | 13 (40.6%) | 56 (50.9%) | 0.31 |
| Hyperlipidemia | 18 (56.3%) | 68 (61.8%) | 0.57 |
| Diabetes mellitus | 11 (34.4%) | 27 (24.5%) | 0.27 |
| Atrial fibrillation | 14 (43.8%) | 40 (36.4%) | 0.45 |
| History of HFrEF | 23 (71.9%) | 59 (53.6%) | 0.07 |
| Previous VT ablation | 11 (34.4%) | 43 (39.1%) | 0.63 |
| VT storm on presentation | 13 (40.6%) | 35 (31.8%) | 0.35 |
| Pre-ablation LVEF (%) | 34.3±11.7 | 46.4±15.2 | <0.001 |
| Pre-ablation LVEDD (mm) | 59.8±10.0 | 56.9±9.1 | 0.16 |
| Pre-ablation LVEDV (mL) | 183.5±68.4 | 160.1±58.4 | 0.17 |
| Pre-ablation LVESD (mm) | 49.2±12.9 | 43.9±11.9 | 0.07 |
| Pre-ablation LVESV (mL) | 119.4±49.5 | 98.0±57.0 | 0.32 |
| Pre-ablation ACEi/ARB/ARNI | 13 (40.6%) | 38 (34.5%) | 0.53 |
| Pre-ablation beta-blocker | 16 (50.0%) | 40 (36.4%) | 0.17 |
| Pre-ablation MRA | 8 (25.0%) | 19 (17.3%) | 0.33 |
| Pre-ablation ‘triple therapy’* | 7 (30.4%) | 7 (11.9%) | 0.05 |
| Post-ablation ACEi/ARB/ARNI | 13 (40.6%) | 38 (34.5%) | 0.53 |
| Post-ablation beta-blocker | 17 (53.1%) | 43 (39.1%) | 0.16 |
| Post-ablation MRA | 9 (28.1%) | 24 (21.8%) | 0.46 |
| Post-ablation ‘triple therapy’* | 7 (30.4%) | 11 (18.6%) | 0.25 |
| VT inducibility | 26 (81.3%) | 102 (92.7%) | 0.06 |
| Radiofrequency time (min) | 51.0±38.9 | 50.9±33.0 | 0.99 |
| Procedure time (min) | 253.4±79.8 | 238.4±86.9 | 0.52 |
| Fluoroscopy time (min) | 15.8±6.8 | 18.9±10.9 | 0.29 |
| Inferior LV ablation | 17 (53.1%) | 40 (36.4%) | 0.09 |
| Septal LV ablation | 9 (28.1%) | 46 (41.8%) | 0.16 |
| Anterior LV ablation | 6 (18.8%) | 21 (19.1%) | 0.97 |
| Lateral LV ablation | 4 (12.5%) | 9 (8.2%) | 0.46 |
| Inferior RV ablation | 2 (6.3%) | 11 (10.0%) | 0.52 |
| Septal RV ablation | 2 (6.3%) | 21 (19.1%) | 0.08 |
| Anterior RV ablation | 0 (0.0%) | 0 (0.0%) | - |
| Lateral RV ablation | 0 (0.0%) | 7 (6.4%) | 0.14 |
| LVOT ablation | 2 (6.3%) | 2 (1.8%) | 0.18 |
| RVOT ablation | 2 (6.3%) | 17 (15.5%) | 0.18 |
| Epicardial ablation | 4 (12.5%) | 7 (6.4%) | 0.25 |

*Values are expressed as n(%) for categorical variables, and mean ± SD for continuous variables. *Pre-ablation and post-ablation ‘triple therapy’ were analysed only in the eligible subgroup of patients with structural heart disease and heart failure with reduced ejection fraction (n=16 for decline in LVEF > 5%, n=66 for no decline in LVEF > 5%).*

**Abbreviations:** ACEi, angiotensin-converting enzyme inhibitor; ARB, angiotensin receptor blocker; ARNI, angiotensin receptor-neprilysin inhibitor; CABG, coronary artery bypass graft; HFrEF, heart failure with reduced ejection fraction; LV, left ventricle; LVOT, left ventricular outflow tract; LVEDD, left ventricular end-diastolic diameter; LVEDV, left ventricular end-diastolic volume; LVEF, left ventricular ejection fraction; LVESD, left-ventricular end-systolic diameter; LVESV, left-ventricular end-systolic volume; MRA, mineralocorticoid receptor antagonist; RV, right ventricle; RVOT, right ventricular outflow tract; VT, ventricular tachycardia.

**Supplementary Table 3. Univariate predictors of VA recurrence following ablation.**

| Variables | VA recurrence (n=72) | No VA recurrence (n=70) | *P*-value |
| --- | --- | --- | --- |
| Age (years) | 61.0±14.5 | 67.3±12.4 | 0.01 |
| Gender (male) | 60 (83.3%) | 63 (90.0%) | 0.24 |
| Ischemic cardiomyopathy | 29 (40.3%) | 41 (58.6%) | 0.03 |
| Previous CABG | 12 (16.7%) | 18 (25.7%) | 0.19 |
| Chronic kidney disease | 10 (13.9%) | 13 (18.6%) | 0.45 |
| Hypertension | 31 (43.1%) | 38 (54.3%) | 0.18 |
| Hyperlipidemia | 39 (54.2%) | 47 (67.1%) | 0.11 |
| Diabetes mellitus | 20 (27.8%) | 18 (25.7%) | 0.78 |
| Atrial fibrillation | 29 (40.3%) | 25 (35.7%) | 0.58 |
| History of HFrEF | 44 (61.1%) | 38 (54.3%) | 0.41 |
| Previous VT ablation | 31 (43.1%) | 23 (32.9%) | 0.21 |
| VT storm on presentation | 19 (26.4%) | 29 (41.4%) | 0.06 |
| Pre-ablation LVEF (%) | 43.1±14.9 | 44.2±15.8 | 0.65 |
| Pre-ablation LVEDD (mm) | 58.7±8.2 | 56.5±10.2 | 0.20 |
| Pre-ablation LVEDV (mL) | 170.1±59.2 | 160.7±63.7 | 0.52 |
| Pre-ablation LVESD (mm) | 45.9±11.0 | 44.0±13.4 | 0.41 |
| Pre-ablation LVESV (mL) | 118.5±58.7 | 92.8±51.8 | 0.17 |
| Pre-ablation ACEi/ARB/ARNI | 25 (34.7%) | 26 (37.1%) | 0.76 |
| Pre-ablation beta-blocker | 28 (38.9%) | 28 (40.0%) | 0.89 |
| Pre-ablation MRA | 14 (19.4%) | 13 (18.6%) | 0.90 |
| Pre-ablation ‘triple therapy’* | 6 (13.6%) | 8 (21.1%) | 0.37 |
| Post-ablation ACEi/ARB/ARNI | 26 (36.1%) | 25 (35.7%) | 0.96 |
| Post-ablation beta-blocker | 29 (40.3%) | 31 (44.3%) | 0.63 |
| Post-ablation MRA | 18 (25.0%) | 15 (21.4%) | 0.61 |
| Post-ablation ‘triple therapy’* | 9 (20.5%) | 9 (23.7%) | 0.73 |
| VT inducibility | 64 (88.9%) | 64 (91.4%) | 0.61 |
| Radiofrequency time (min) | 62.7±41.2 | 43.8±27.6 | 0.04 |
| Procedure time (min) | 266.0±83.8 | 226.1±82.8 | 0.05 |
| Fluoroscopy time (min) | 18.4±7.7 | 17.8±11.1 | 0.84 |
| Procedure success^¶^ |  |  | 0.02 |
| Complete | 27 (41.5%) | 41 (64.1%) |  |
| Partial | 31 (47.7%) | 21 (32.8%) |  |
| Unsuccessful | 7 (10.8%) | 2 (3.1%) |  |
| Inferior LV ablation | 25 (34.7%) | 32 (45.7%) | 0.18 |
| Septal LV ablation | 29 (40.3%) | 26 (37.1%) | 0.70 |
| Anterior LV ablation | 15 (20.8%) | 12 (17.1%) | 0.58 |
| Lateral LV ablation | 6 (8.3%) | 7 (10.0%) | 0.73 |
| Inferior RV ablation | 6 (8.3%) | 7 (10.0%) | 0.73 |
| Septal RV ablation | 7 (9.7%) | 16 (22.9%) | 0.03 |
| Anterior RV ablation | 0 (0.0%) | 0 (0.0%) | - |
| Lateral RV ablation | 5 (6.9%) | 2 (2.9%) | 0.26 |
| LVOT ablation | 0 (0.0%) | 4 (5.7%) | 0.04 |
| RVOT ablation | 10 (13.9%) | 9 (12.9%) | 0.86 |
| Epicardial ablation | 7 (9.7%) | 4 (5.7%) | 0.37 |
| Post-ablation LVEF increase >5% | 10 (13.9%) | 22 (31.4%) | 0.01 |

*Values are expressed as n(%) for categorical variables, and mean ± SD for continuous variables. *Pre-ablation and post-ablation ‘triple therapy’ were analysed only in the eligible subgroup of patients with structural heart disease and heart failure with reduced ejection fraction (n=14 for VA recurrence, n=68 for no VA recurrence). ^¶^Procedural success was analysed only in the subgroup of patients where VA was inducible (n=65 for VA recurrence, n=64 for no VA recurrence).*

**Abbreviations:** ACEi, angiotensin-converting enzyme inhibitor; ARB, angiotensin receptor blocker; ARNI, angiotensin receptor-neprilysin inhibitor; CABG, coronary artery bypass graft; HFrEF, heart failure with reduced ejection fraction; LV, left ventricle; LVOT, left ventricular outflow tract; LVEDD, left ventricular end-diastolic diameter; LVEDV, left ventricular end-diastolic volume; LVEF, left ventricular ejection fraction; LVESD, left-ventricular end-systolic diameter; LVESV, left-ventricular end-systolic volume; MRA, mineralocorticoid receptor antagonist; RV, right ventricle; RVOT, right ventricular outflow tract; VA, ventricular arrhythmia; VT, ventricular tachycardia.

**Supplementary Table 4. Multivariate predictors of VA recurrence following ablation**.

| Variables | Odds ratio | 95% confidence interval | *P*-value |
| --- | --- | --- | --- |
| Patient’s age | 0.96 | 0.93 – 0.99 | 0.04 |
| Ischaemic cardiomyopathy | 0.80 | 0.33 – 1.94 | 0.62 |
| Procedure success |  |  |  |
| Complete^¶^ |  |  | 0.03 |
| Partial | 2.62 | 1.10 – 6.22 | 0.03 |
| Unsuccessful | 5.94 | 0.98 – 35.92 | 0.04 |
| Ablation in septal RV | 0.18 | 0.06 – 0.57 | 0.01 |
| Post-ablation LVEF increase >5% | 0.30 | 0.11 – 0.82 | 0.02 |
| Ablation in LVOT | *NS** | *NS** | *NS** |

**The variable ‘Ablation in LVOT’ was not statistically significant (P=1.00), with an unspecified upper bound for the 95% confidence interval. ^¶^For procedural success, ‘complete’ was used as the reference category.*

**Abbreviations:** LVOT, left ventricular outflow tract; NS, not significant; RV, right ventricle; VA, ventricular arrhythmia.
